# Supplementary material for: Leveraging learned representations and multitask learning for lysine methylation site discovery
Source: bioRxiv. 2025 Sep 21:2025.08.27.672583. Originally published 2025 Sep 1. Preprint. [Version 2] doi: 10.1101/2025.08.27.672583 (PMC12424735; doi:10.1101/2025.08.27.672583)
Supplement: Supplement 1 [file media-1.pdf]

Leveraging learned representations and multitask learning for lysine methylation site discovery

François Charih, Mullen Boulter, Kyle K. Biggar and James R. Green

Table S.1: Isolation list of 100 putative methylation sites identified with MethylSight 2.0 and selected for *in vitro* validation

	Protein (Uniprot ID)	Position	Score	Peptide	Modified	Methyl state	m/z (+2)	m/z (+3)	m/z (+4)
1	A0A024R1R8	8	0.962	MSSHEGGK	False	null	416.685	278.125	208.846
2	A0A024R1R8	8	0.962	MSSHEGGK	False	mono	423.692	282.797	212.35
3	A0A024R1R8	8	0.962	MSSHEGGK	False	di	430.7	287.469	215.854
4	A0A024R1R8	8	0.962	MSSHEGGK	False	tri	437.708	292.141	219.358
5	A2PYH4	1395	0.914	NPNSSNYK	False	null	462.215	308.479	231.611
6	A2PYH4	1395	0.914	NPNSSNYK	False	mono	469.222	313.151	235.115
7	A2PYH4	1395	0.914	NPNSSNYK	False	di	476.23	317.823	238.619
8	A2PYH4	1395	0.914	NPNSSNYK	False	tri	483.238	322.494	242.123
9	A4D1E9	376	0.935	QLLNLWISDTMSSTEPPSK	False	null	1074.038	716.361	537.523
10	A4D1E9	376	0.935	QLLNLWISDTMSSTEPPSK	False	mono	1081.046	721.033	541.027
11	A4D1E9	376	0.935	QLLNLWISDTMSSTEPPSK	False	di	1088.054	725.705	544.531
12	A4D1E9	376	0.935	QLLNLWISDTMSSTEPPSK	False	tri	1095.062	730.377	548.034
13	B2CW77	75	0.957	VSLVGELSK	False	null	466.277	311.187	233.642
14	B2CW77	75	0.957	VSLVGELSK	False	mono	473.284	315.859	237.146
15	B2CW77	75	0.957	VSLVGELSK	False	di	480.292	320.531	240.65
16	B2CW77	75	0.957	VSLVGELSK	False	tri	487.3	325.202	244.154
17	O00300	255	0.912	QHSSQEQTFQLLK	False	null	787.402	525.27	394.205
18	O00300	255	0.912	QHSSQEQTFQLLK	False	mono	794.41	529.942	397.709
19	O00300	255	0.912	QHSSQEQTFQLLK	False	di	801.418	534.614	401.213
20	O00300	255	0.912	QHSSQEQTFQLLK	False	tri	808.426	539.286	404.716
21	O14966	20	0.923	VLVVGDAAVGK	True	null	514.311	343.21	257.659
22	O14966	20	0.923	VLVVGDAAVGK	True	mono	521.319	347.882	261.163
23	O14966	20	0.923	VLVVGDAAVGK	True	di	528.327	352.553	264.667
24	O14966	20	0.923	VLVVGDAAVGK	True	tri	535.334	357.225	268.171
25	O15144	256	0.909	DYLHYHIK	True	null	544.78	363.522	272.894
26	O15144	256	0.909	DYLHYHIK	True	mono	551.788	368.194	276.397
27	O15144	256	0.909	DYLHYHIK	True	di	558.795	372.866	279.901
28	O15144	256	0.909	DYLHYHIK	True	tri	565.803	377.538	283.405
29	O43505	394	0.942	EAENQHNK	True	null	485.223	323.818	243.115
30	O43505	394	0.942	EAENQHNK	True	mono	492.231	328.49	246.619
31	O43505	394	0.942	EAENQHNK	True	di	499.239	333.161	250.123
32	O43505	394	0.942	EAENQHNK	True	tri	506.246	337.833	253.627
33	O75129	1337	0.917	NTYGESK	False	null	399.685	266.792	200.346
34	O75129	1337	0.917	NTYGESK	False	mono	406.693	271.464	203.85
35	O75129	1337	0.917	NTYGESK	False	di	413.701	276.136	207.354
36	O75129	1337	0.917	NTYGESK	False	tri	420.709	280.808	210.858
37	O75223	181	0.976	VSEEIEDIIK	False	null	587.814	392.212	294.41
38	O75223	181	0.976	VSEEIEDIIK	False	mono	594.822	396.883	297.914
39	O75223	181	0.976	VSEEIEDIIK	False	di	601.829	401.555	301.418
40	O75223	181	0.976	VSEEIEDIIK	False	tri	608.837	406.227	304.922

	Protein (Uniprot ID)	Position	Score	Peptide	Modified	Methyl state	m/z (+2)	m/z (+3)	m/z (+4)
41	O75608	105	0.913	ALIDQEVK	True	null	458.261	305.843	229.634
42	O75608	105	0.913	ALIDQEVK	True	mono	465.269	310.515	233.138
43	O75608	105	0.913	ALIDQEVK	True	di	472.277	315.187	236.642
44	O75608	105	0.913	ALIDQEVK	True	tri	479.284	319.859	240.146
45	O75934	218	0.932	QQHGEANK	True	null	456.22	304.483	228.614
46	O75934	218	0.932	QQHGEANK	True	mono	463.228	309.154	232.118
47	O75934	218	0.932	QQHGEANK	True	di	470.236	313.826	235.622
48	O75934	218	0.932	QQHGEANK	True	tri	477.244	318.498	239.125
49	O95372	69	0.918	IPVTLNMK	False	null	458.27	305.849	229.639
50	O95372	69	0.918	IPVTLNMK	False	mono	465.278	310.521	233.143
51	O95372	69	0.918	IPVTLNMK	False	di	472.286	315.193	236.647
52	O95372	69	0.918	IPVTLNMK	False	tri	479.294	319.865	240.15
53	P02794	120	0.964	NVNQSLLELHK	True	null	647.859	432.242	324.433
54	P02794	120	0.964	NVNQSLLELHK	True	mono	654.867	436.914	327.937
55	P02794	120	0.964	NVNQSLLELHK	True	di	661.875	441.586	331.441
56	P02794	120	0.964	NVNQSLLELHK	True	tri	668.883	446.258	334.945
57	P06276	586	0.934	WNNYMMDWK	False	null	644.268	429.848	322.638
58	P06276	586	0.934	WNNYMMDWK	False	mono	651.276	434.52	326.142
59	P06276	586	0.934	WNNYMMDWK	False	di	658.284	439.191	329.645
60	P06276	586	0.934	WNNYMMDWK	False	tri	665.291	443.863	333.149
61	P08590	107	0.967	QEELNTK	False	null	431.219	287.815	216.113
62	P08590	107	0.967	QEELNTK	False	mono	438.227	292.487	219.617
63	P08590	107	0.967	QEELNTK	False	di	445.235	297.159	223.121
64	P08590	107	0.967	QEELNTK	False	tri	452.243	301.831	226.625
65	P0C0P6	80	0.987	NGVGTGMK	False	null	382.192	255.13	191.6
66	P0C0P6	80	0.987	NGVGTGMK	False	mono	389.2	259.802	195.104
67	P0C0P6	80	0.987	NGVGTGMK	False	di	396.208	264.474	198.607
68	P0C0P6	80	0.987	NGVGTGMK	False	tri	403.215	269.146	202.111
69	P0DME0	13	0.962	QSPLPLQK	False	null	455.771	304.183	228.389
70	P0DME0	13	0.962	QSPLPLQK	False	mono	462.779	308.855	231.893
71	P0DME0	13	0.962	QSPLPLQK	False	di	469.787	313.527	235.397
72	P0DME0	13	0.962	QSPLPLQK	False	tri	476.795	318.199	238.901
73	P0DP57	66	0.922	VLSNTEDLPLVTK	False	null	714.901	476.936	357.954
74	P0DP57	66	0.922	VLSNTEDLPLVTK	False	mono	721.909	481.608	361.458
75	P0DP57	66	0.922	VLSNTEDLPLVTK	False	di	728.917	486.28	364.962
76	P0DP57	66	0.922	VLSNTEDLPLVTK	False	tri	735.924	490.952	368.466
77	P11233	159	0.955	AEQWNVNYVETSAK	True	null	819.892	546.93	410.449
78	P11233	159	0.955	AEQWNVNYVETSAK	True	mono	826.899	551.602	413.953
79	P11233	159	0.955	AEQWNVNYVETSAK	True	di	833.907	556.274	417.457
80	P11233	159	0.955	AEQWNVNYVETSAK	True	tri	840.915	560.946	420.961
81	P13073	78	0.907	ASWSSLSMDEK	False	null	620.779	414.189	310.893
82	P13073	78	0.907	ASWSSLSMDEK	False	mono	627.787	418.861	314.397
83	P13073	78	0.907	ASWSSLSMDEK	False	di	634.795	423.532	317.901
84	P13073	78	0.907	ASWSSLSMDEK	False	tri	641.803	428.204	321.405
85	P20472	37	0.975	FFQMVGLK	False	null	485.265	323.846	243.136

	Protein (Uniprot ID)	Position	Score	Peptide	Modified	Methyl state	m/z (+2)	m/z (+3)	m/z (+4)
86	P20472	37	0.975	FFQMVGLK	False	mono	492.273	328.518	246.64
87	P20472	37	0.975	FFQMVGLK	False	di	499.281	333.189	250.144
88	P20472	37	0.975	FFQMVGLK	False	tri	506.288	337.861	253.648
89	P25686	59	0.934	EVAEAYEVLSDK	True	null	676.833	451.558	338.92
90	P25686	59	0.934	EVAEAYEVLSDK	True	mono	683.84	456.229	342.424
91	P25686	59	0.934	EVAEAYEVLSDK	True	di	690.848	460.901	345.928
92	P25686	59	0.934	EVAEAYEVLSDK	True	tri	697.856	465.573	349.432
93	P25705	539	0.947	ISEQSDAK	True	null	439.217	293.147	220.112
94	P25705	539	0.947	ISEQSDAK	True	mono	446.225	297.819	223.616
95	P25705	539	0.947	ISEQSDAK	True	di	453.232	302.491	227.12
96	P25705	539	0.947	ISEQSDAK	True	tri	460.24	307.163	230.624
97	P25787	50	0.957	AANGVVLATEK	True	null	536.803	358.205	268.905
98	P25787	50	0.957	AANGVVLATEK	True	mono	543.811	362.877	272.409
99	P25787	50	0.957	AANGVVLATEK	True	di	550.819	367.549	275.913
100	P25787	50	0.957	AANGVVLATEK	True	tri	557.827	372.22	279.417
101	P26447	18	0.947	ALDVMVSTFHK	True	null	624.326	416.553	312.667
102	P26447	18	0.947	ALDVMVSTFHK	True	mono	631.334	421.225	316.171
103	P26447	18	0.947	ALDVMVSTFHK	True	di	638.342	425.897	319.675
104	P26447	18	0.947	ALDVMVSTFHK	True	tri	645.35	430.569	323.179
105	P26641	428	0.936	EYFSWEGAFQHVGK	True	null	842.891	562.263	421.949
106	P26641	428	0.936	EYFSWEGAFQHVGK	True	mono	849.899	566.935	425.453
107	P26641	428	0.936	EYFSWEGAFQHVGK	True	di	856.907	571.607	428.957
108	P26641	428	0.936	EYFSWEGAFQHVGK	True	tri	863.915	576.279	432.461
109	P27216	284	0.963	AEVDLQGIK	False	null	486.772	324.85	243.889
110	P27216	284	0.963	AEVDLQGIK	False	mono	493.779	329.522	247.393
111	P27216	284	0.963	AEVDLQGIK	False	di	500.787	334.194	250.897
112	P27216	284	0.963	AEVDLQGIK	False	tri	507.795	338.866	254.401
113	P27348	157	0.97	QTIDNSQGAYQEAFDISK	True	null	1007.971	672.317	504.489
114	P27348	157	0.97	QTIDNSQGAYQEAFDISK	True	mono	1014.979	676.988	507.993
115	P27348	157	0.97	QTIDNSQGAYQEAFDISK	True	di	1021.987	681.66	511.497
116	P27348	157	0.97	QTIDNSQGAYQEAFDISK	True	tri	1028.995	686.332	515.001
117	P28340	1007	0.942	VGGLLAFAK	True	null	438.271	292.516	219.639
118	P28340	1007	0.942	VGGLLAFAK	True	mono	445.279	297.188	223.143
119	P28340	1007	0.942	VGGLLAFAK	True	di	452.287	301.86	226.647
120	P28340	1007	0.942	VGGLLAFAK	True	tri	459.295	306.532	230.151
121	P30084	101	0.972	AFAAGADIK	True	null	432.235	288.492	216.621
122	P30084	101	0.972	AFAAGADIK	True	mono	439.243	293.164	220.125
123	P30084	101	0.972	AFAAGADIK	True	di	446.25	297.836	223.629
124	P30084	101	0.972	AFAAGADIK	True	tri	453.258	302.508	227.133
125	P46734	340	0.937	TDIAAFVK	True	null	432.745	288.832	216.876
126	P46734	340	0.937	TDIAAFVK	True	mono	439.753	293.504	220.38
127	P46734	340	0.937	TDIAAFVK	True	di	446.761	298.176	223.884
128	P46734	340	0.937	TDIAAFVK	True	tri	453.768	302.848	227.388
129	P49137	371	0.947	VDYEQIK	True	null	447.732	298.824	224.37
130	P49137	371	0.947	VDYEQIK	True	mono	454.74	303.496	227.874

	Protein (Uniprot ID)	Position	Score	Peptide	Modified	Methyl state	m/z (+2)	m/z (+3)	m/z (+4)
131	P49137	371	0.947	VDYEQIK	True	di	461.748	308.168	231.377
132	P49137	371	0.947	VDYEQIK	True	tri	468.755	312.839	234.881
133	P51809	172	0.94	TENLVDSSVTFK	True	null	670.341	447.23	335.674
134	P51809	172	0.94	TENLVDSSVTFK	True	mono	677.348	451.901	339.178
135	P51809	172	0.94	TENLVDSSVTFK	True	di	684.356	456.573	342.682
136	P51809	172	0.94	TENLVDSSVTFK	True	tri	691.364	461.245	346.186
137	P56470	83	0.906	VVFNTLQGGK	True	null	531.801	354.87	266.404
138	P56470	83	0.906	VVFNTLQGGK	True	mono	538.809	359.541	269.908
139	P56470	83	0.906	VVFNTLQGGK	True	di	545.816	364.213	273.412
140	P56470	83	0.906	VVFNTLQGGK	True	tri	552.824	368.885	276.916
141	P58546	11	0.916	EFMWALK	True	null	462.736	308.826	231.872
142	P58546	11	0.916	EFMWALK	True	mono	469.744	313.498	235.376
143	P58546	11	0.916	EFMWALK	True	di	476.752	318.17	238.879
144	P58546	11	0.916	EFMWALK	True	tri	483.76	322.842	242.383
145	P59998	166	0.969	IVAEEFLK	True	null	474.774	316.852	237.89
146	P59998	166	0.969	IVAEEFLK	True	mono	481.781	321.523	241.394
147	P59998	166	0.969	IVAEEFLK	True	di	488.789	326.195	244.898
148	P59998	166	0.969	IVAEEFLK	True	tri	495.797	330.867	248.402
149	P60228	407	0.906	LGHVVMGNNAVSPYQQVIEK	True	null	1092.068	728.381	546.537
150	P60228	407	0.906	LGHVVMGNNAVSPYQQVIEK	True	mono	1099.075	733.053	550.041
151	P60228	407	0.906	LGHVVMGNNAVSPYQQVIEK	True	di	1106.083	737.725	553.545
152	P60228	407	0.906	LGHVVMGNNAVSPYQQVIEK	True	tri	1113.091	742.396	557.049
153	P61244	24	0.956	FQSAADK	True	null	383.69	256.129	192.349
154	P61244	24	0.956	FQSAADK	True	mono	390.698	260.801	195.853
155	P61244	24	0.956	FQSAADK	True	di	397.706	265.473	199.357
156	P61244	24	0.956	FQSAADK	True	tri	404.714	270.145	202.86
157	P61758	59	0.971	LDEQYQK	True	null	462.227	308.487	231.617
158	P61758	59	0.971	LDEQYQK	True	mono	469.235	313.159	235.121
159	P61758	59	0.971	LDEQYQK	True	di	476.243	317.831	238.625
160	P61758	59	0.971	LDEQYQK	True	tri	483.251	322.503	242.129
161	P98175	915	0.942	GSSYGVTSTESYK	False	null	683.312	455.877	342.16
162	P98175	915	0.942	GSSYGVTSTESYK	False	mono	690.32	460.549	345.664
163	P98175	915	0.942	GSSYGVTSTESYK	False	di	697.328	465.221	349.167
164	P98175	915	0.942	GSSYGVTSTESYK	False	tri	704.336	469.893	352.671
165	Q08623	123	0.93	HGIPFALATSSGSASFDMK	False	null	962.467	641.98	481.737
166	Q08623	123	0.93	HGIPFALATSSGSASFDMK	False	mono	969.475	646.652	485.241
167	Q08623	123	0.93	HGIPFALATSSGSASFDMK	False	di	976.483	651.324	488.745
168	Q08623	123	0.93	HGIPFALATSSGSASFDMK	False	tri	983.491	655.996	492.249
169	Q0VDE8	75	0.944	GTLHGQEK	False	null	435.227	290.487	218.117
170	Q0VDE8	75	0.944	GTLHGQEK	False	mono	442.235	295.159	221.621
171	Q0VDE8	75	0.944	GTLHGQEK	False	di	449.243	299.831	225.125
172	Q0VDE8	75	0.944	GTLHGQEK	False	tri	456.251	304.503	228.629
173	Q13043	480	0.964	QPILDAIEAK	False	null	549.314	366.545	275.16
174	Q13043	480	0.964	QPILDAIEAK	False	mono	556.322	371.217	278.664
175	Q13043	480	0.964	QPILDAIEAK	False	di	563.329	375.889	282.168

	Protein (Uniprot ID)	Position	Score	Peptide	Modified	Methyl state	m/z (+2)	m/z (+3)	m/z (+4)
176	Q13043	480	0.964	QPILDAIEAK	False	tri	570.337	380.561	285.672
177	Q13823	721	0.933	TNDSEGQK	False	null	439.696	293.467	220.352
178	Q13823	721	0.933	TNDSEGQK	False	mono	446.704	298.138	223.856
179	Q13823	721	0.933	TNDSEGQK	False	di	453.712	302.81	227.36
180	Q13823	721	0.933	TNDSEGQK	False	tri	460.72	307.482	230.863
181	Q14914	318	0.955	EYIIEGFENMPAAFMGMLK	False	null	1096.017	731.014	548.512
182	Q14914	318	0.955	EYIIEGFENMPAAFMGMLK	False	mono	1103.025	735.686	552.016
183	Q14914	318	0.955	EYIIEGFENMPAAFMGMLK	False	di	1110.033	740.358	555.52
184	Q14914	318	0.955	EYIIEGFENMPAAFMGMLK	False	tri	1117.041	745.03	559.024
185	Q15286	189	0.969	QQQQQQNDVVK	True	null	671.839	448.229	336.423
186	Q15286	189	0.969	QQQQQQNDVVK	True	mono	678.847	452.9	339.927
187	Q15286	189	0.969	QQQQQQNDVVK	True	di	685.855	457.572	343.431
188	Q15286	189	0.969	QQQQQQNDVVK	True	tri	692.863	462.244	346.935
189	Q15506	49	0.897	EQPDNIPAFAAAYFESLLEK	False	null	1127.057	751.707	564.032
190	Q15506	49	0.897	EQPDNIPAFAAAYFESLLEK	False	mono	1134.065	756.379	567.536
191	Q15506	49	0.897	EQPDNIPAFAAAYFESLLEK	False	di	1141.073	761.051	571.04
192	Q15506	49	0.897	EQPDNIPAFAAAYFESLLEK	False	tri	1148.081	765.723	574.544
193	Q16836	249	0.9	EDIDTAMK	True	null	461.713	308.144	231.36
194	Q16836	249	0.9	EDIDTAMK	True	mono	468.721	312.816	234.864
195	Q16836	249	0.9	EDIDTAMK	True	di	475.729	317.488	238.368
196	Q16836	249	0.9	EDIDTAMK	True	tri	482.736	322.16	241.872
197	Q32NC0	85	0.96	NYTLSFK	False	null	436.729	291.489	218.868
198	Q32NC0	85	0.96	NYTLSFK	False	mono	443.737	296.16	222.372
199	Q32NC0	85	0.96	NYTLSFK	False	di	450.745	300.832	225.876
200	Q32NC0	85	0.96	NYTLSFK	False	tri	457.753	305.504	229.38
201	Q5MAI5	33	0.93	TSGQVVAVK	True	null	444.761	296.843	222.884
202	Q5MAI5	33	0.93	TSGQVVAVK	True	mono	451.769	301.515	226.388
203	Q5MAI5	33	0.93	TSGQVVAVK	True	di	458.777	306.187	229.892
204	Q5MAI5	33	0.93	TSGQVVAVK	True	tri	465.785	310.859	233.396
205	Q5TC84	344	0.918	MSSPLASSHNSQTSMHK	False	null	915.417	610.614	458.212
206	Q5TC84	344	0.918	MSSPLASSHNSQTSMHK	False	mono	922.425	615.286	461.716
207	Q5TC84	344	0.918	MSSPLASSHNSQTSMHK	False	di	929.433	619.958	465.22
208	Q5TC84	344	0.918	MSSPLASSHNSQTSMHK	False	tri	936.441	624.629	468.724
209	Q5VT25	1647	0.954	SSAQNGSALK	False	null	481.749	321.502	241.378
210	Q5VT25	1647	0.954	SSAQNGSALK	False	mono	488.757	326.173	244.882
211	Q5VT25	1647	0.954	SSAQNGSALK	False	di	495.764	330.845	248.386
212	Q5VT25	1647	0.954	SSAQNGSALK	False	tri	502.772	335.517	251.89
213	Q6DD88	399	0.958	QLALDHFK	False	null	486.269	324.515	243.638
214	Q6DD88	399	0.958	QLALDHFK	False	mono	493.277	329.187	247.142
215	Q6DD88	399	0.958	QLALDHFK	False	di	500.285	333.859	250.646
216	Q6DD88	399	0.958	QLALDHFK	False	tri	507.293	338.531	254.15
217	Q6IQ20	18	0.906	MDENESNQSLMTSSQYPK	False	null	1044.946	696.966	522.977
218	Q6IQ20	18	0.906	MDENESNQSLMTSSQYPK	False	mono	1051.954	701.638	526.481
219	Q6IQ20	18	0.906	MDENESNQSLMTSSQYPK	False	di	1058.962	706.31	529.984
220	Q6IQ20	18	0.906	MDENESNQSLMTSSQYPK	False	tri	1065.97	710.982	533.488

	Protein (Uniprot ID)	Position	Score	Peptide	Modified	Methyl state	m/z (+2)	m/z (+3)	m/z (+4)
221	Q6P2I3	18	0.929	LLTALLQAQK	False	null	549.848	366.901	275.427
222	Q6P2I3	18	0.929	LLTALLQAQK	False	mono	556.856	371.573	278.931
223	Q6P2I3	18	0.929	LLTALLQAQK	False	di	563.863	376.245	282.435
224	Q6P2I3	18	0.929	LLTALLQAQK	False	tri	570.871	380.917	285.939
225	Q7Z6K5	203	0.958	TGASWTDNIMAQK	True	null	711.838	474.894	356.423
226	Q7Z6K5	203	0.958	TGASWTDNIMAQK	True	mono	718.846	479.566	359.926
227	Q7Z6K5	203	0.958	TGASWTDNIMAQK	True	di	725.853	484.238	363.43
228	Q7Z6K5	203	0.958	TGASWTDNIMAQK	True	tri	732.861	488.91	366.934
229	Q86SX6	151	0.93	LGIHSALLDEK	False	null	598.338	399.228	299.672
230	Q86SX6	151	0.93	LGIHSALLDEK	False	mono	605.346	403.899	303.176
231	Q86SX6	151	0.93	LGIHSALLDEK	False	di	612.353	408.571	306.68
232	Q86SX6	151	0.93	LGIHSALLDEK	False	tri	619.361	413.243	310.184
233	Q8IX90	389	0.954	YNSNLATPIAIK	False	null	652.864	435.578	326.936
234	Q8IX90	389	0.954	YNSNLATPIAIK	False	mono	659.872	440.25	330.44
235	Q8IX90	389	0.954	YNSNLATPIAIK	False	di	666.88	444.922	333.944
236	Q8IX90	389	0.954	YNSNLATPIAIK	False	tri	673.888	449.594	337.447
237	Q8IY31	34	0.923	VLDPEVTQQTIELK	True	null	806.943	538.298	403.975
238	Q8IY31	34	0.923	VLDPEVTQQTIELK	True	mono	813.951	542.97	407.479
239	Q8IY31	34	0.923	VLDPEVTQQTIELK	True	di	820.959	547.642	410.983
240	Q8IY31	34	0.923	VLDPEVTQQTIELK	True	tri	827.967	552.314	414.487
241	Q8N118	504	0.938	NGMYLHLK	False	null	488.258	325.841	244.632
242	Q8N118	504	0.938	NGMYLHLK	False	mono	495.265	330.513	248.136
243	Q8N118	504	0.938	NGMYLHLK	False	di	502.273	335.185	251.64
244	Q8N118	504	0.938	NGMYLHLK	False	tri	509.281	339.857	255.144
245	Q8N4H5	46	0.979	VTPFILK	True	null	409.263	273.178	205.135
246	Q8N4H5	46	0.979	VTPFILK	True	mono	416.271	277.849	208.639
247	Q8N4H5	46	0.979	VTPFILK	True	di	423.278	282.521	212.143
248	Q8N4H5	46	0.979	VTPFILK	True	tri	430.286	287.193	215.647
249	Q8N8Q3	170	0.905	LLQVDGLENNALHK	True	null	782.428	521.954	391.718
250	Q8N8Q3	170	0.905	LLQVDGLENNALHK	True	mono	789.436	526.626	395.222
251	Q8N8Q3	170	0.905	LLQVDGLENNALHK	True	di	796.444	531.298	398.726
252	Q8N8Q3	170	0.905	LLQVDGLENNALHK	True	tri	803.452	535.97	402.229
253	Q8NA97	84	0.91	LFIYSSK	False	null	429.242	286.497	215.125
254	Q8NA97	84	0.91	LFIYSSK	False	mono	436.25	291.169	218.629
255	Q8NA97	84	0.91	LFIYSSK	False	di	443.258	295.841	222.132
256	Q8NA97	84	0.91	LFIYSSK	False	tri	450.265	300.513	225.636
257	Q8NE09	1254	0.942	ASSSTMSLK	False	null	456.229	304.488	228.618
258	Q8NE09	1254	0.942	ASSSTMSLK	False	mono	463.237	309.16	232.122
259	Q8NE09	1254	0.942	ASSSTMSLK	False	di	470.244	313.832	235.626
260	Q8NE09	1254	0.942	ASSSTMSLK	False	tri	477.252	318.504	239.13
261	Q8NEF9	378	0.942	SLDFPQNEPQIK	True	null	708.362	472.577	354.685
262	Q8NEF9	378	0.942	SLDFPQNEPQIK	True	mono	715.37	477.249	358.189
263	Q8NEF9	378	0.942	SLDFPQNEPQIK	True	di	722.378	481.921	361.692
264	Q8NEF9	378	0.942	SLDFPQNEPQIK	True	tri	729.385	486.593	365.196
265	Q8NFR3	66	0.909	LAWEFFSK	False	null	514.266	343.18	257.637

	Protein (Uniprot ID)	Position	Score	Peptide	Modified	Methyl state	m/z (+2)	m/z (+3)	m/z (+4)
266	Q8NFR3	66	0.909	LAWEFFSK	False	mono	521.274	347.852	261.141
267	Q8NFR3	66	0.909	LAWEFFSK	False	di	528.282	352.524	264.644
268	Q8NFR3	66	0.909	LAWEFFSK	False	tri	535.289	357.195	268.148
269	Q8NGY0	325	0.975	MMGNTVALK	False	null	482.751	322.17	241.879
270	Q8NGY0	325	0.975	MMGNTVALK	False	mono	489.759	326.842	245.383
271	Q8NGY0	325	0.975	MMGNTVALK	False	di	496.767	331.514	248.887
272	Q8NGY0	325	0.975	MMGNTVALK	False	tri	503.775	336.186	252.391
273	Q8WUD6	383	0.926	HLHLNIFK	False	null	511.301	341.203	256.154
274	Q8WUD6	383	0.926	HLHLNIFK	False	mono	518.309	345.875	259.658
275	Q8WUD6	383	0.926	HLHLNIFK	False	di	525.316	350.547	263.162
276	Q8WUD6	383	0.926	HLHLNIFK	False	tri	532.324	355.219	266.666
277	Q92499	702	0.952	AAGGGSYK	True	null	355.677	237.454	178.342
278	Q92499	702	0.952	AAGGGSYK	True	mono	362.685	242.126	181.846
279	Q92499	702	0.952	AAGGGSYK	True	di	369.693	246.798	185.35
280	Q92499	702	0.952	AAGGGSYK	True	tri	376.701	251.469	188.854
281	Q92901	297	0.952	GPHMEDGK	False	null	435.692	290.797	218.35
282	Q92901	297	0.952	GPHMEDGK	False	mono	442.7	295.469	221.854
283	Q92901	297	0.952	GPHMEDGK	False	di	449.708	300.141	225.358
284	Q92901	297	0.952	GPHMEDGK	False	tri	456.716	304.813	228.862
285	Q96CQ1	174	0.924	VYQTDGLK	False	null	462.245	308.499	231.626
286	Q96CQ1	174	0.924	VYQTDGLK	False	mono	469.253	313.171	235.13
287	Q96CQ1	174	0.924	VYQTDGLK	False	di	476.261	317.843	238.634
288	Q96CQ1	174	0.924	VYQTDGLK	False	tri	483.269	322.515	242.138
289	Q96IG2	408	0.897	THLPNIK	False	null	411.745	274.833	206.376
290	Q96IG2	408	0.897	THLPNIK	False	mono	418.753	279.504	209.88
291	Q96IG2	408	0.897	THLPNIK	False	di	425.761	284.176	213.384
292	Q96IG2	408	0.897	THLPNIK	False	tri	432.769	288.848	216.888
293	Q96IM9	158	0.925	MPQEINYK	False	null	511.752	341.504	256.38
294	Q96IM9	158	0.925	MPQEINYK	False	mono	518.76	346.176	259.884
295	Q96IM9	158	0.925	MPQEINYK	False	di	525.768	350.848	263.388
296	Q96IM9	158	0.925	MPQEINYK	False	tri	532.776	355.52	266.892
297	Q96KB5	8	0.984	MEGISNFK	True	null	463.226	309.153	232.117
298	Q96KB5	8	0.984	MEGISNFK	True	mono	470.234	313.825	235.621
299	Q96KB5	8	0.984	MEGISNFK	True	di	477.242	318.497	239.124
300	Q96KB5	8	0.984	MEGISNFK	True	tri	484.25	323.169	242.628
301	Q96PM5	239	0.934	STVQFHILGMK	False	null	630.842	420.897	315.925
302	Q96PM5	239	0.934	STVQFHILGMK	False	mono	637.85	425.569	319.429
303	Q96PM5	239	0.934	STVQFHILGMK	False	di	644.858	430.241	322.933
304	Q96PM5	239	0.934	STVQFHILGMK	False	tri	651.866	434.913	326.436
305	Q96QD9	261	0.976	TAVPSFLTK	True	null	482.279	321.855	241.643
306	Q96QD9	261	0.976	TAVPSFLTK	True	mono	489.287	326.527	245.147
307	Q96QD9	261	0.976	TAVPSFLTK	True	di	496.295	331.199	248.651
308	Q96QD9	261	0.976	TAVPSFLTK	True	tri	503.303	335.871	252.155
309	Q99757	147	0.93	NGDVVDK	False	null	373.688	249.461	187.347
310	Q99757	147	0.93	NGDVVDK	False	mono	380.695	254.133	190.851

	Protein (Uniprot ID)	Position	Score	Peptide	Modified	Methyl state	m/z (+2)	m/z (+3)	m/z (+4)
311	Q99757	147	0.93	NGDVVDK	False	di	387.703	258.805	194.355
312	Q99757	147	0.93	NGDVVDK	False	tri	394.711	263.476	197.859
313	Q9BSD7	73	0.923	VGLEPPPGK	True	null	447.258	298.508	224.133
314	Q9BSD7	73	0.923	VGLEPPPGK	True	mono	454.266	303.18	227.637
315	Q9BSD7	73	0.923	VGLEPPPGK	True	di	461.274	307.852	231.141
316	Q9BSD7	73	0.923	VGLEPPPGK	True	tri	468.282	312.524	234.644
317	Q9BTT4	82	0.915	NPQLYTK	True	null	432.235	288.492	216.621
318	Q9BTT4	82	0.915	NPQLYTK	True	mono	439.243	293.164	220.125
319	Q9BTT4	82	0.915	NPQLYTK	True	di	446.25	297.836	223.629
320	Q9BTT4	82	0.915	NPQLYTK	True	tri	453.258	302.508	227.133
321	Q9BV29	98	0.913	GLNQEVTSK	True	null	488.259	325.842	244.633
322	Q9BV29	98	0.913	GLNQEVTSK	True	mono	495.267	330.514	248.137
323	Q9BV29	98	0.913	GLNQEVTSK	True	di	502.275	335.185	251.641
324	Q9BV29	98	0.913	GLNQEVTSK	True	tri	509.282	339.857	255.145
325	Q9BVG4	63	0.939	LISSVDPQFLK	False	null	623.856	416.24	312.431
326	Q9BVG4	63	0.939	LISSVDPQFLK	False	mono	630.864	420.911	315.935
327	Q9BVG4	63	0.939	LISSVDPQFLK	False	di	637.871	425.583	319.439
328	Q9BVG4	63	0.939	LISSVDPQFLK	False	tri	644.879	430.255	322.943
329	Q9BZE2	407	0.961	QTSAFVEGVK	False	null	533.282	355.857	267.145
330	Q9BZE2	407	0.961	QTSAFVEGVK	False	mono	540.29	360.529	270.649
331	Q9BZE2	407	0.961	QTSAFVEGVK	False	di	547.298	365.201	274.153
332	Q9BZE2	407	0.961	QTSAFVEGVK	False	tri	554.306	369.873	277.657
333	Q9H3Q1	114	0.973	DSALFVK	True	null	390.219	260.481	195.613
334	Q9H3Q1	114	0.973	DSALFVK	True	mono	397.226	265.153	199.117
335	Q9H3Q1	114	0.973	DSALFVK	True	di	404.234	269.825	202.621
336	Q9H3Q1	114	0.973	DSALFVK	True	tri	411.242	274.497	206.125
337	Q9H7S9	141	0.897	SAPGAASAAAALK	True	null	543.301	362.537	272.154
338	Q9H7S9	141	0.897	SAPGAASAAAALK	True	mono	550.309	367.208	275.658
339	Q9H7S9	141	0.897	SAPGAASAAAALK	True	di	557.317	371.88	279.162
340	Q9H7S9	141	0.897	SAPGAASAAAALK	True	tri	564.325	376.552	282.666
341	Q9HAV7	138	0.923	DDNPHLK	False	null	419.706	280.14	210.357
342	Q9HAV7	138	0.923	DDNPHLK	False	mono	426.714	284.812	213.861
343	Q9HAV7	138	0.923	DDNPHLK	False	di	433.722	289.484	217.365
344	Q9HAV7	138	0.923	DDNPHLK	False	tri	440.73	294.156	220.869
345	Q9NS73	208	0.965	TDAIFTPYPGFK	True	null	678.845	452.899	339.926
346	Q9NS73	208	0.965	TDAIFTPYPGFK	True	mono	685.853	457.571	343.43
347	Q9NS73	208	0.965	TDAIFTPYPGFK	True	di	692.861	462.243	346.934
348	Q9NS73	208	0.965	TDAIFTPYPGFK	True	tri	699.869	466.915	350.438
349	Q9NSI8	68	0.915	TSNNGGGLGK	True	null	452.728	302.154	226.868
350	Q9NSI8	68	0.915	TSNNGGGLGK	True	mono	459.736	306.826	230.371
351	Q9NSI8	68	0.915	TSNNGGGLGK	True	di	466.743	311.498	233.875
352	Q9NSI8	68	0.915	TSNNGGGLGK	True	tri	473.751	316.17	237.379
353	Q9NUV7	530	0.908	EMLDTVLEALDEMGDLLQLK	False	null	1138.574	759.385	569.79
354	Q9NUV7	530	0.908	EMLDTVLEALDEMGDLLQLK	False	mono	1145.581	764.057	573.294
355	Q9NUV7	530	0.908	EMLDTVLEALDEMGDLLQLK	False	di	1152.589	768.729	576.798

	Protein (Uniprot ID)	Position	Score	Peptide	Modified	Methyl state	m/z (+2)	m/z (+3)	m/z (+4)
356	Q9NUV7	530	0.908	EMLDTVLEALDEMGDLLQLK	False	tri	1159.597	773.401	580.302
357	Q9NV56	200	0.977	VLTANSNPSSPSAAK	True	null	722.376	481.919	361.691
358	Q9NV56	200	0.977	VLTANSNPSSPSAAK	True	mono	729.383	486.591	365.195
359	Q9NV56	200	0.977	VLTANSNPSSPSAAK	True	di	736.391	491.263	368.699
360	Q9NV56	200	0.977	VLTANSNPSSPSAAK	True	tri	743.399	495.935	372.203
361	Q9NWQ9	128	0.916	QLEFSEPDFVAK	True	null	705.351	470.57	353.179
362	Q9NWQ9	128	0.916	QLEFSEPDFVAK	True	mono	712.359	475.242	356.683
363	Q9NWQ9	128	0.916	QLEFSEPDFVAK	True	di	719.367	479.914	360.187
364	Q9NWQ9	128	0.916	QLEFSEPDFVAK	True	tri	726.374	484.585	363.691
365	Q9NX63	173	0.924	AAEEVEAK	True	null	423.714	282.812	212.361
366	Q9NX63	173	0.924	AAEEVEAK	True	mono	430.722	287.484	215.864
367	Q9NX63	173	0.924	AAEEVEAK	True	di	437.729	292.155	219.368
368	Q9NX63	173	0.924	AAEEVEAK	True	tri	444.737	296.827	222.872
369	Q9NXW2	177	0.913	QYDQFGDDK	True	null	558.236	372.493	279.621
370	Q9NXW2	177	0.913	QYDQFGDDK	True	mono	565.243	377.165	283.125
371	Q9NXW2	177	0.913	QYDQFGDDK	True	di	572.251	381.837	286.629
372	Q9NXW2	177	0.913	QYDQFGDDK	True	tri	579.259	386.508	290.133
373	Q9P0L0	24	0.917	HEQILVLDPPTDLK	True	null	809.446	539.966	405.227
374	Q9P0L0	24	0.917	HEQILVLDPPTDLK	True	mono	816.454	544.638	408.731
375	Q9P0L0	24	0.917	HEQILVLDPPTDLK	True	di	823.462	549.31	412.234
376	Q9P0L0	24	0.917	HEQILVLDPPTDLK	True	tri	830.469	553.982	415.738
377	Q9UBS4	66	0.931	NPDDPQAQEK	True	null	571.26	381.176	286.133
378	Q9UBS4	66	0.931	NPDDPQAQEK	True	mono	578.267	385.847	289.637
379	Q9UBS4	66	0.931	NPDDPQAQEK	True	di	585.275	390.519	293.141
380	Q9UBS4	66	0.931	NPDDPQAQEK	True	tri	592.283	395.191	296.645
381	Q9UF47	184	0.933	DVDFPVFLQPTNANEK	False	null	917.455	611.972	459.231
382	Q9UF47	184	0.933	DVDFPVFLQPTNANEK	False	mono	924.462	616.644	462.735
383	Q9UF47	184	0.933	DVDFPVFLQPTNANEK	False	di	931.47	621.316	466.239
384	Q9UF47	184	0.933	DVDFPVFLQPTNANEK	False	tri	938.478	625.988	469.743
385	Q9UHD4	213	0.946	HAVEGAEQWQQK	False	null	705.842	470.897	353.424
386	Q9UHD4	213	0.946	HAVEGAEQWQQK	False	mono	712.849	475.569	356.928
387	Q9UHD4	213	0.946	HAVEGAEQWQQK	False	di	719.857	480.241	360.432
388	Q9UHD4	213	0.946	HAVEGAEQWQQK	False	tri	726.865	484.913	363.936
389	Q9ULV4	19	0.931	HVFGQAVK	True	null	443.251	295.836	222.129
390	Q9ULV4	19	0.931	HVFGQAVK	True	mono	450.259	300.508	225.633
391	Q9ULV4	19	0.931	HVFGQAVK	True	di	457.266	305.18	229.137
392	Q9ULV4	19	0.931	HVFGQAVK	True	tri	464.274	309.852	232.641
393	Q9Y3V2	267	0.899	LFSEFVLALVK	False	null	633.379	422.588	317.193
394	Q9Y3V2	267	0.899	LFSEFVLALVK	False	mono	640.387	427.26	320.697
395	Q9Y3V2	267	0.899	LFSEFVLALVK	False	di	647.394	431.932	324.201
396	Q9Y3V2	267	0.899	LFSEFVLALVK	False	tri	654.402	436.604	327.705
397	Q9Y6G3	114	0.935	VEHLEEGPMIEQLSK	True	null	869.938	580.294	435.472
398	Q9Y6G3	114	0.935	VEHLEEGPMIEQLSK	True	mono	876.945	584.966	438.976
399	Q9Y6G3	114	0.935	VEHLEEGPMIEQLSK	True	di	883.953	589.638	442.48
400	Q9Y6G3	114	0.935	VEHLEEGPMIEQLSK	True	tri	890.961	594.31	445.984
